# Establishing a digital health platform in an academic medical center supporting rural communities

**DOI:** 10.1017/cts.2020.11

**Published:** 2020-04-28

**Authors:** Anita Walden, Aaron S. Kemp, Linda J. Larson-Prior, Thomas Kim, Jennifer Gan, Hannah McCoy, Nalin Payakachat, Wendy Ward, Hari Eswaran

**Affiliations:** 1 Department of Biomedical Informatics, University of Arkansas for Medical Sciences, Little Rock, AR, USA; 2 Psychiatry, University of Arkansas for Medical Sciences, Little Rock, AR, USA; 3 Neurology, Neurobiology & Developmental Sciences, University of Arkansas for Medical Sciences, Little Rock, AR, USA; 4 Radiation Oncology, University of Arkansas for Medical Sciences, Little Rock, AR, USA; 5 Center for Health Literacy, University of Arkansas for Medical Sciences, Little Rock, AR, USA; 6 Institute of Digital Health and Innovation, University of Arkansas for Medical Sciences, Little Rock, AR, USA; 7 College of Pharmacy, University of Arkansas for Medical Sciences, Little Rock, AR, USA; 8 Interprofessional Faculty Development, University of Arkansas for Medical Sciences, Little Rock, AR, USA

**Keywords:** Digital health, digital technology, mobile health, innovation, rural health

## Abstract

The University of Arkansas for Medical Sciences (UAMS), like many rural states, faces clinical and research obstacles to which digital innovation is seen as a promising solution. To implement digital technology, a mobile health interest group was established to lay the foundation for an enterprise-wide digital health innovation platform. To create a foundation, an interprofessional team was established, and a series of formal networking events was conducted. Three online digital health training models were developed, and a full-day regional conference was held featuring nationally recognized speakers and panel discussions with clinicians, researchers, and patient advocates involved in digital health programs at UAMS. Finally, an institution-wide survey exploring the interest in and knowledge of digital health technologies was distributed. The networking events averaged 35–45 attendees. About 100 individuals attended the regional conference with positive feedback from participants. To evaluate mHealth knowledge at the institution, a survey was completed by 257 UAMS clinicians, researchers, and staff. It revealed that there are opportunities to increase training, communication, and collaboration for digital health implementation. The inclusion of the mobile health working group in the newly formed Institute for Digital Health and Innovation provides a nexus for healthcare providers and researches to facilitate translational research.

## Introduction

Digital health, including technologies from telehealth to wearables, is seen as an important aspect of modern healthcare [1]. This reflects an interest in decreasing costs by providing both interventional and preventative care, a shift from hospital and clinic-based care to home-based care, and the need to serve populations in areas remote from standard hospital or clinic-based care. While deployed in both urban and rural areas, these technologies may have the most significant impact in rural and medically underserved areas. Rural populations are commonly medically underserved due to rural hospital closures, few physicians, and even fewer specialists [2]. Furthermore, these populations are generally faced with barriers to access due to transportation challenges, distance from medical centers, and lack of insurance that limits their ability to access care centered in more urban areas. In the USA, residents of rural counties are more likely to exhibit poor heath behaviors (e.g., higher rates of smoking), greater all-cause morbidity and mortality, lower socioeconomic status, and lower levels of clinical care than in urban counties [3]. These problems are further exacerbated by low levels of health literacy, which make it more difficult for individuals to access publicly available health information [4]. The promise of digital health is in improving access to care, providing preventative healthcare to individuals with environmental, economic, and health literacy barriers to access, and improving research in new digital health technologies that can further increase the health span in a chronically underserved population. To achieve these goals, academic medical centers (AMCs) will be the bridge to the future in the digital transformation of medical practice [5,6].

New and practical methods of real-world data collection and exchange can facilitate translational research to bridge the gap between research, clinical care, the community, and the individual patient [5, 7]. AMCs have the leadership and technical capabilities to influence and drive these changes, while serving as test beds for new innovations [8, 9]. However, both digital health research and the integration of digital technology into the healthcare delivery system are complex, requiring skilled and experienced personnel, and the knowledge of regulatory requirements [10]. To successfully evaluate and implement emerging platforms, AMCs need a visionary, structured, systematic framework [11] supported by cross-disciplinary teams that cover multiple aspects of digital health to meet institutional and areal goals. This is particularly true where the focus is on rural areas, and logistical hurdles to implementation are often extensive. Several AMCs have recognized these needs and have established digital institutes or centers that foster education, skill building, and cross-disciplinary team building to support quality care delivery solutions and research [12, 13].

The University of Arkansas for Medical Sciences (UAMS), the state’s only AMC, provides health services to a rural state that ranks 47th in health status [14]. UAMS has had a strong presence in digital health since the development of a statewide telehealth program in 2003 [15–18]. This initial telehealth program is a statewide consultative service for family practitioners, obstetricians, neonatologists, and pediatricians with the mission to improve treatment of high-risk pregnancies through a Medicaid-funded, patient-centered approach that brings high-risk obstetrical services to rural hospitals through real-time, telehealth technologies [15, 17]. This program, the Antenatal and Neonatal Guidelines, Education and Learning System (ANGELS) program, has consulted high-risk pregnant women at 44 rural sites, decreased postpartum complications, and contributed to a decrease in the 60-day infant mortality rate in Arkansas [15, 18]. Since then, the state has made tremendous progress in telehealth programs focused on stroke, spinal cord, and traumatic brain injury [18]. In stroke, the Arkansas Stroke Assistance through Virtual Emergency Support (AR-SAVES) program is a Medicaid-funded effort that connects potential stroke victims in rural emergency departments with neurologists at urban hospitals in Arkansas [18]. In 2019, these telemedicine programs were formally rolled into the Institute for Digital Health and Innovation (IDHI) (idhi.uams.edu).

As a leader in the deployment of telehealth to rural and underserved populations, to take full advantage of current digital health technologies, there was a need to incorporate new methods such as mobile technology, social media, remote-based monitoring, wearables, and other innovative solutions to improve access to care and research. To address this need, a mobile health multi-disciplinary interest group formed in 2016 that consisted primarily of UAMS clinicians and researchers. Their purpose was to identify the current use of digital technology within the institution and to establish an enterprise-wide foundation for a digital health innovation platform to implement and support digital innovations. This group pursued the goal of fostering cross-stakeholder collaboration, providing educational opportunities and supporting researchers and clinicians interested in adopting meaningful, secure, and quality digital health efforts. Early efforts led to the clear realization that researchers and clinicians often lacked the expertise to successfully implement these technologies. Barriers to development of new digital health technologies included data access, ownership of data when working with external vendors, and challenges with implementation and validation.

This paper describes the process which one AMC provided the groundwork for development of an enterprise-wide digital health innovation platform to serve researchers, clinicians, and patients in a rural state. We describe the methods and approaches used to determine the degree to which digital health technologies were needed or currently utilized at UAMS, the degree to which entrepreneurs and the UAMS community interacted to develop such technologies, and the level of interest in the clinical, technological, and research communities in the development and deployment of these technologies.

## Approach

### Mobile Health (mHealth) Interest Group

An initial group of four collaborators from the Departments of Biomedical Informatics, Psychiatry, the Center for Distance Health and Radiation Oncology at UAMS met to build on the success of the statewide UAMS telemedicine programs to explore the use of cutting-edge digital technologies in a rural state. The group was supported by member departments and the UAMS Translational Research Institute. The focus of the group was to develop and implement an institutional framework of collaboration, education and training, information sharing, and process development.

### Interprofessional Collaboration

To establish the framework, it was important to identify, bring together, and harmonize efforts of those with an interest in using digital technology. The interest group formed an interprofessional team of clinicians, researchers, informaticists, a bioethicist, lawyers, technology investment experts, and educators from UAMS and area universities to accomplish that goal. The initial task was to identify those using or with an interest in using digital technology, foster collaboration, and provide an avenue for connecting with other technologists in the community.

### Education and Outreach

Four approaches to education and outreach were implemented: (1) three networking events designed to support collaborations between community technology innovators, the UAMS technology transfer office (Bioventures), and UAMS faculty in development of digital health technologies; (2) development and deployment of three online training modules covering commercialization, design, and execution of validation studies, and relevant regulatory, legal, and security considerations; (3) a full-day regional conference featuring nationally recognized speakers and panel discussions between clinicians, researchers, and patient advocates involved in digital health programs at UAMS; and (4) an institution-wide survey on digital health technology familiarity and use.

### Networking Events

To launch institutional awareness of the newly formed mobile health working group, a poster was presented at a UAMS event showcasing medical discoveries. This was followed by two formal networking events, called mHealth Mingles, that were hosted by the local office for technology transfers at UAMS (BioVentures). Brief presentations from clinicians and researchers using digital health technologies at UAMS and local technology developers seeking collaborative development or clinical validation partners provided brief presentations followed by open question periods for participants. These events provided a critically important opportunity to promote collaborative development and validation projects between UAMS clinicians or researchers and local technology developers.

### Online Training Modules

Three online learning modules were developed by the mHealth working group that provided didactic content on relevant aspects of designing, developing, and validating digital health technologies and intellectual property. Working in collaborations with the UAMS Center for Distance Health (CDH) and the South Central Telehealth Resource Center, provided the resources for final deployment of these online modules, which are described below (https://learntelehealth.org/course/digital-health-training-module).

1. *Commercialization as a Catalyst for Innovations in Digital Health*: This training module provides healthcare entities and individuals with information on how commercialization can serve as a catalyst for digital health innovation. The target audience includes healthcare administrators, researchers, and providers. The specific learning objectives are to (A) understand the need for the healthcare industry to adapt to the changing demands of healthcare consumers by developing and/or leveraging new technologies to efficiently transform patient-centric data sources into clinically meaningful information that optimizes treatment outcomes, supports shared clinical decisions, and/or decreases costs of care; (B) recognize the potential of funding the development, validation, and clinical implementation of mobile health and wearable biomonitoring technologies using private and public sources of support for small business ventures; and (C) identify local support services to help protect intellectual property, articulate the specific use-case scenario and value proposition to relevant stakeholders, and execute well-designed validation field studies to demonstrate that value.
2. *Clinical Validation and Testing in Digital Health*: The purpose of this training module is to help healthcare entities and individuals using digital health and wearable technologies with validation and field testing prior to implementation in their research and clinical programs. The target audience for this module includes healthcare administration, researchers, and providers. The specific learning objectives are to (A) identify three types of measurable outcomes for evaluating clinical utility and effectiveness of digital health tools and technologies; (B) determine optimal contexts for the validation of digital health tools; and (C) distinguish relevant regulatory factors related to the clinical validation and implementation of digital health tools.
3. *Regulatory Considerations in Digital Health*: The purpose of this training module is to help healthcare entities and individuals using digital health and wearable technologies in their research and clinical programs to recognize the relevant regulatory and security issues that must be taken into consideration. The target audience for this module includes healthcare administration, researchers, and providers. The specific learning objectives are to (A) understand how to apply Health Insurance Portability and Accountability Act regulations to cloud technologies; (B) identify allowable prerequisites of patient health identifiers when using digital health technologies in clinical and research settings; (C) develop techniques to evaluate and apply technical, practical, and legal solutions when working with patient data in the context of digital health technology applications.

Each of the online training modules includes a brief content quiz to assess retention of the information presented.

### Full-Day Digital Health Conference

A full-day Digital Health Conference was organized to share ideas and experiences pertaining to digital health innovation in research and clinical settings. Presenters included nationally recognized speakers from funding agencies (e.g., Patient-Centered Outcomes Research Institute), local experts in the use of technologies to reach rural or underserved populations, and panel discussions featuring clinicians, researchers, and patient advocates from the local community. Attendees to the Digital Health Conference completed a brief survey regarding their perceptions of the events in which they participated. An additional survey was distributed to all UAMS faculty and researchers to assess interest in and overall experience with digital health technologies.

## Metrics and Outcomes

### Integration of MHealth Interest Group

In 2019, UAMS created the IDHI. Creation of this Institute acknowledged the strong presence of telehealth at UAMS and pointed to the institutional commitment to expand its digital health footprint. The mHealth interest group joined the IDHI soon after its inception, where it will continue to serve the institution and community in research, education, and development of digital health technologies.

### Networking Events

The two mHealth mingles attracted 35–45 attendees, including faculty and staff from across the University of Arkansas system, technology vendors from the community, city agencies, and technology incubators. The events were well received and, as hoped, resulted in a clearer understanding of the current uses of these technologies as well as areas in which clinical and research faculty and staff hoped to innovate. Evaluations indicated that attendees would like to gain greater technical assistance or expertise in the development of digital health technologies for their applications.

### Online Training Modules

At present, no information on module use is available. Learning objectives are clearly stated for each module, and test questions assessing comprehension of the information provided are included. These assessments are for self-learning only and are not tracked.

### Conference Survey

A conference was held to introduce the topic of Digital Health and mHealth and to provide information concerning funding opportunities, reimbursement policies, and current research taking place around campus. Patients from the community were also invited to participate to share their stories and thoughts about the use of digital health. A total of 99 individuals attended the Digital Health Conference. Attendees included researchers, clinicians, patients, study coordinators, informaticists, research assistants, and programmers. Of these, 31 completed a brief survey regarding their perceptions of each of the sessions at the conference. A total of 97% of the attendees said that the session objectives were met and 98% said that they learned something new by attending the conference. Additional comments were solicited and indicated that attendees would like to have another conference that addressed medical care more directly and felt that their participation had stimulated an interest in digital health technologies in their domains of interest. Patient attendees noted a strong interest in increased digital health solutions for rural areas, and more collaboration with the health system on approaches.

### Questionnaire Assessing Digital Health Use at UAMS

**Fig. 1.**
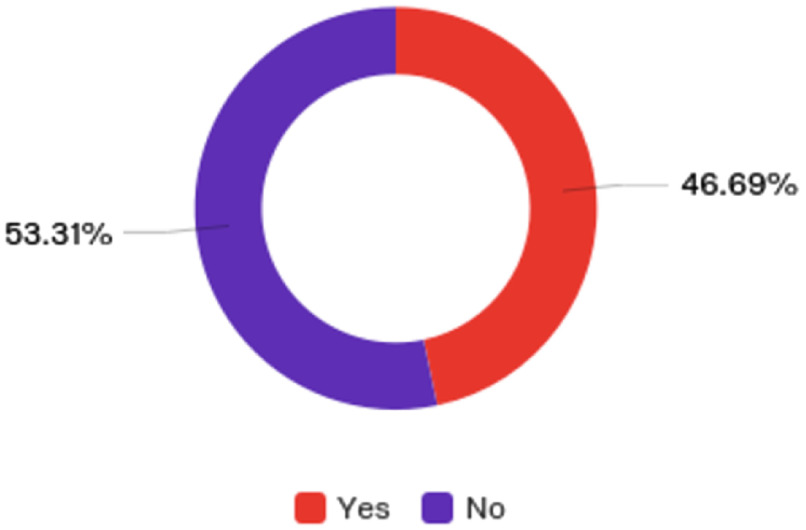
Currently using digital technologies.

After over a year of establishing the foundation for a digital health platform that would systematically assist research teams and clinician, the mHealth working group sent out a questionnaire to evaluate their progress. The Assessing Digital Health Use UAMS Questionnaire was distributed to faculty and staff across UAMS. There were 20 questions that covered use of digital health at UAMS, awareness of digital health programs and resources, and barriers to implementation and needs. Two-hundred and fifty-seven individuals responded. Almost half of respondents have utilized digital technologies (Fig. 1).

The majority of individuals (99%) who responded to the survey were unaware of the multi-stakeholder synergy opportunities called mHealth Mingles, and 93% were unaware of the UAMS Digital Health Conference.

Based on the responses concerning barriers to implementation and needs, many respondents were interested in information on practical approaches to implementation in rural settings as well as opportunities to collaborate (Table 1).

**Table 1.** Opportunities for resources and training

Opportunities for resources and training
Training on digital devices and communication methods, implementation, and deployment More information on wearables and apps use in research and clinical care Approaches to selecting the best device on method Opportunities for collaboration List of tools and devices others have used Practical approaches to starting a digital health project How to implement technologies to rural clinical practice and legal aspects? How to address use of digital technologies for clinical specialties? Skills needed to be successful Resources for funding digital health research and clinical projects

## Discussion

Healthcare services in the USA healthcare show strong disparities in rural locations. Between 2004 and 2014, 179 rural US counties lost hospital obstetric services, resulting in increased preterm and out-of-hospital births [19, 20]. The lack of obstetric services impacts over 27 million women and infants in the USA and represents significant resource and financial burdens. Arkansas addressed this problem using a telemedicine approach, developing video-based obstetrical consultations via the ANGELS program [18]. The program successfully reduced 60-day infant mortality rates in the state by 0.5% between 2003 and 2004. These successes led to the establishment of the CDH in 2006, which continued to expand telehealth services to underserved populations [18].

Arkansas, as a largely rural state, is a fitting location to enhance innovative and personalized remote healthcare services. With an age-adjusted stroke mortality rate of 53.7/100,000 in 2009, among the highest in the nation [21], in 2008 UAMS developed the AR-SAVES telestroke program. The purpose was to improve transport times to medical facilities qualified for stroke care and, as a result, to decrease time to treat. Two primary arms of the program are (1) development of telestroke centers in rural hospitals and (2) provision of information on qualified stroke centers to emergency services paramedics transporting patients [21–23]. Its success is reflected in the approximately 2000 patients treated, a treatment rate increase of >30%, and improved transport times to qualified stroke centers [21, 24].

Taking advantage of the strong telehealth presence in the IDHI, the mHealth working group was formed to provide a platform for collaborations between health professionals and technology specialists to both take advantage of current mobile health technologies and create new ones. The success of these early efforts will be enhanced by the integration of the mHealth working group and the IDHI, which together will provide a platform to develop, evaluate, and deploy new mobile digital health modalities to underserved communities in the state.

Information obtained from participants in the mHealth Mingles, the mHealth Conference, and the institutional survey showed that, institution-wide, surprisingly few healthcare professionals were aware of the educational mechanisms provided to assist them in implementation and use of digital health solutions. This suggests that stronger efforts are needed to increase awareness of the need for MHealth technologies and the educational opportunities available to aid in their development. There was a strong interest in the development of new, easy-to-use, and readily available mHealth technologies, with an equally strong need to provide training platforms for their development, testing, and deployment. This is in keeping with a global assessment by the World Health Organization [25], where one of the four primary barriers to the use of digital health technologies was the lack technical knowledge.

The need for methods that provide high-quality health care to underserved communities while reducing overall costs is well recognized [9]. Despite this clear need, and efforts by several AMCs to implement appropriate programs, such methods are not well integrated with current medical practices in the USA. As we found at our institution, the lack of a clear path to develop digital health technologies beyond telehealth was reflected in uncoordinated and siloed development largely unknown outside of the department or clinic that implemented it. This issue is one that all AMCs must tackle [13] if we are to realize the promise of these technologies to the health of our citizens.

The hope is that these technologies will improve education, research, and delivery of care by enabling data sharing and team-based care approaches across healthcare settings while promoting translation of technologies from bench-to-bedside. This translation will provide an opportunity to deliver health care in low-health resource settings where there is often a lack of diagnostic and monitoring technologies. These goals are needs driven and largely focused on the design of technically advanced health systems where communication of health information is frequently not considered [4]. Two important aspects of communication that were not addressed in our study, but represent a critical component of the utilization of these technologies, are interprofessional education (IPE) for providers and health literacy for communities and patients. IPE is a transformative educational program that creates active learning experiences for learners from diverse health professions to learn new skills, such as digital health [26, 27]. Health literacy, which requires both basic reading, writing, and numeracy skills and the ability to acquire and understand relevant health information, is an important component of the ability to make informed health-related choices [28]. Low health literacy can result in more hospitalizations, poorer overall health, and increased mortality as well as increased medical costs [29, 30]. Thus, incorporation of best practices for health literacy into all digital health technologies is a critical component of their success.

The results of our work suggest that centralizing the informational, research, and practical aspects of digital health technologies is a necessary first step toward creating an environment in which innovative uses of digital health technologies can be discussed, developed, and deployed for the purpose of providing better and less expensive health care to the underserved citizens of largely rural states such as Arkansas.

## Conclusion

A medical center serving a rural state leveraged its strengths in telemedicine to include mHealth technologies in the newly established IDHI. Feedback from the community showed is a need and a desire for education, training and better dissemination of information to successfully implement and manage digital health technology in health care and research. The new institute will address those needs and provide an environment to foster the use of digital health innovation supporting translational research and quality clinical care across a rural state.

## References

[1] Dorsey ER , Topol EJ . State of telehealth. New England Journal of Medicine 2016; 375(2): 154–161. 27410924 10.1056/NEJMra1601705

[2] Fordyce MA , et al. 2005 physician supply and distribution in rural areas of the United States. Final Report 2007; 116.

[3] Anderson TJ , et al. A cross-sectional study on health differences between rural and non-rural US counties using the County Health Rankings. BMC Health Services Research 2015; 15(1): 441. 26423746 10.1186/s12913-015-1053-3 PMC4590732

[4] Kreps GL . Achieving the promise of digital health information systems. Journal of Public Health Research 2014; 3(3): 471. 25553317 10.4081/jphr.2014.471 PMC4274501

[5] Van der Laan AL , Boenink M . Beyond bench and bedside: disentangling the concept of translational research. Health Care Analysis 2015; 23(1): 32–49. 23248053 10.1007/s10728-012-0236-x PMC4293498

[6] Dzau VJ , et al. The role of academic health science systems in the transformation of medicine. The Lancet 2010; 375 (9718): 949–953. 10.1016/S0140-6736(09)61082-5 19800111

[7] Rubio DM , et al. Defining translational research: implications for training. Academic Medicine: Journal of the Association of American Medical Colleges 2010; 85(3): 470. 20182120 10.1097/ACM.0b013e3181ccd618 PMC2829707

[8] DePasse JW , et al. (eds.). Academic medical centers as digital health catalysts. *Healthcare* 2014: Elsevier. 10.1016/j.hjdsi.2014.05.006 26250503

[9] Ellner AL , et al. Health systems innovation at academic health centers: leading in a new era of health care delivery. Academic Medicine 2015; 90(7): 872–880. 25738387 10.1097/ACM.0000000000000679

[10] Sharma A , et al. Using digital health technology to better generate evidence and deliver evidence-based care. Journal of the American College of Cardiology 2018; 71(23): 2680–2690. 29880129 10.1016/j.jacc.2018.03.523

[11] Khuntia J , et al. The University of Colorado Digital Health Consortium Initiative: A collaborative model of education, research and service. Journal of Commercial Biotechnology 2014; 20(3).

[12] Al Kuwaiti A , Al Muhanna FA , Al Amri S . Implementation of digital health technology at academic medical centers in Saudi Arabia. Oman Medical Journal 2018; 33(5): 367. 30210714 10.5001/omj.2018.69 PMC6131920

[13] Mann DM , et al. Building digital innovation capacity at a large academic medical center. npj Digital Medicine 2019; 2(1): 13. 31304362 10.1038/s41746-019-0088-y PMC6550180

[14] Foundation UH. America’s Health Rankings 2018 [Internet]. (https://www.americashealthrankings.org/learn/reports/2018-annual-report)

[15] Hall RW , Hall-Barrow J , Garcia-Rill E . Neonatal regionalization through telemedicine using a community based research and education core facility. Ethnicity & Disease 2010; 20(1 Suppl. 1): S1-136–S1-140. PMC3323108 20521402

[16] Lowery C , et al. ANGELS and University of Arkansas for Medical Sciences paradigm for distant obstetrical care delivery. American Journal of Obstetrics and Gynecology 2007; 196(6): 534.e1–534.e9. 17547884 10.1016/j.ajog.2007.01.027

[17] Bronstein JM , et al. Improving perinatal regionalization for preterm deliveries in a Medicaid covered population: initial impact of the Arkansas ANGELS intervention. Health Services Research 2011; 46(4): 1082–1103. 21413980 10.1111/j.1475-6773.2011.01249.x PMC3165179

[18] Lowery CL , et al. Distributing medical expertise: the evolution and impact of telemedicine in arkansas. Health Affairs 2014; 33(2): 235–243. 24493766 10.1377/hlthaff.2013.1001

[19] Hung P , et al. Access to obstetric services in rural counties still declining, with 9 percent losing services, 2004–14. Health Affairs 2017; 36(9): 1663–1671. 28874496 10.1377/hlthaff.2017.0338

[20] Kozhimannil KB , et al. Association between loss of hospital-based obstetric services and birth outcomes in rural counties in the United States. JAMA 2018; 319(12): 1239–1247. 29522161 10.1001/jama.2018.1830 PMC5885848

[21] Brown AT , et al. Emergency transport of stroke suspects in a rural state: opportunities for improvement. The American Journal of Emergency Medicine 2016; 34(8): 1640–1644. 27344100 10.1016/j.ajem.2016.06.044 PMC4998736

[22] Nalleballe K , et al. Ideal telestroke time targets: telestroke-based treatment times in the United States stroke belt. *Journal of Telemedicine and Telecare* 2018: 1357633X18805661. 10.1177/1357633X18805661 30352525

[23] Brown AT , et al. Abstract TP306: mobile application,“WeTrain911” to decrease stroke mortality by enhancing emergency dispatcher training. Stroke 2018; 49(Suppl. 1): ATP306–ATP.

[24] Lowery C (ed.). Telemedicine approaches to advancing health equity for rural trauma patients. APHA’s 2018 Annual Meeting & Expo (Nov 10–Nov 14); 2018: American Public Health Association.

[25] Kay M , Santos J , Takane M . MHealth: New horizons for health through mobile technologies. World Health Organization 2011; 64(7): 66–71.

[26] Lutfiyya MN , Brandt BF , Cerra F . Reflections from the intersection of health professions education and clinical practice: the state of the science of interprofessional education and collaborative practice. Academic Medicine 2016; 91(6): 766–771. 26959223 10.1097/ACM.0000000000001139

[27] Schmitt M , et al. Core competencies for interprofessional collaborative practice: reforming health care by transforming health professionals’ education. Academic Medicine 2011; 86(11): 1351. 22030650 10.1097/ACM.0b013e3182308e39

[28] Nutbeam D , McGill B , Premkumar P . Improving health literacy in community populations: a review of progress. Health Promotion International 2017; 33(5): 901–911. 10.1093/heapro/dax015 28369557

[29] Haun JN , et al. Association between health literacy and medical care costs in an integrated healthcare system: a regional population based study. BMC Health Services Research 2015; 15(1): 249. 26113118 10.1186/s12913-015-0887-z PMC4482196

[30] Mackert M , et al. Health literacy and health information technology adoption: the potential for a new digital divide. Journal of Medical Internet Research 2016; 18(10): e264. 27702738 10.2196/jmir.6349 PMC5069402

